# Prevalence of Nontuberculous Mycobacterial Pulmonary Disease, Germany, 2009–2014

**DOI:** 10.3201/eid2206.151642

**Published:** 2016-06

**Authors:** Felix C. Ringshausen, Dirk Wagner, Andrés de Roux, Roland Diel, David Hohmann, Lennart Hickstein, Tobias Welte, Jessica Rademacher

**Affiliations:** German Center for Lung Research, Giessen, Germany (F.C. Ringshausen, R. Diel, T. Welte);; Hannover Medical School, Hannover, Germany (F.C. Ringshausen, T. Welte, J. Rademacher);; University Hospital Freiburg, Freiburg, Germany (D. Wagner);; Pneumologische Praxis am Schloss Charlottenburg, Berlin, Germany (A. de Roux);; University Medical Center Schleswig-Holstein, Kiel, Germany (R. Diel);; HGC GesundheitsConsult, Düsseldorf, Germany (D. Hohmann);; Health Risk Institute, Berlin (L. Hickstein)

**Keywords:** Atypical mycobacterial infection, bronchiectasis, COPD, NTM-PD, chronic obstructive pulmonary disease, cystic fibrosis, international classification of diseases, Mycobacterium avium complex, nontuberculous mycobacteria, pulmonary, respiratory infections, tuberculosis, Germany

## Abstract

We analyzed routine statutory health insurance claim data to determine prevalence of nontuberculous mycobacterial pulmonary disease in Germany. Documented prevalence rates of this nonnotifiable disease increased from 2.3 to 3.3 cases/100,000 population from 2009 to 2014. Prevalence showed a strong association with advanced age and chronic obstructive pulmonary disease.

Nontuberculous mycobacteria (NTM) are a biologically diverse group of microorganisms that may cause progressive NTM pulmonary disease (NTM-PD), particularly in persons who have risk factors such as advanced age or chronic airway diseases (*1*–*3*). However, population-based data on the epidemiology of NTM-PD are scarce, particularly in Europe. We used hospital discharge codes from the International Classification of Diseases*,* 10th revision (ICD-10), as a surrogate to demonstrate that NTM-PD prevalence is steadily increasing in Germany, as is the case in many other countries (*4*–*6*). However, we assumed that most patients with this chronic infection are managed in outpatient care. The objective of this population-based study was to estimate the annual overall prevalence rates of NTM-PD in Germany over a 6-year period and to analyze the distribution of age and sex, the site of healthcare provision, and concomitant conditions, using a representative sample of routine statutory health insurance claims data.

## The Study

This study was based on the population of Germany of ≈81 million during 2009–2014, and a subgroup of ≈70 million persons (86%) covered by German statutory health insurance (Table 1) (*7*). Because this study was based on anonymous routine data, institutional review board approval and patient consent were not required. We obtained anonymous health claims data from the Health Risk Institute health services research database, which contains a subset of ≈7 million persons covered by statutory health insurance. This database has been validated and shows good overall accuracy in documenting the German population in terms of illness, death, and drug usage; in addition, the number of insured persons in the database remains high over time (*8*). We established data characteristics by adjustments for age and sex according to the distribution within the general population of Germany (*7*). Only persons who were continuously insured within the respective year were considered. Of those persons, we obtained a 5% sample representative of the German population for each year of the study period, consisting of ≈4 million persons per year (Table 1). From these samples, we extracted records that used the ICD-10 diagnosis code A31.0 (NTM-PD) for either a primary or a secondary diagnosis, and we analyzed data according to age, sex, the site of healthcare provision (Table 1), and the most common and relevant associated conditions, as represented by concomitantly recorded ICD-10 codes (Table 2).

**Table 1:** Prevelance of nontuberculous mycobacterial pulmonary disease, stratified by age, sex, and year; Germany, 2009–2014*

Characteristic	2009	2010	2011	2012	2013	2014
Population of Germany, total no.	81,802,257	81,751,602	80,327,900	80,523,746	80,767,463	81,197,537
Mean age, y	43.4	43.7	43.9	44.1	44.2	44.3
Sex ratio, F:M	1.04	1.04	1.05	1.05	1.04	1.04
No. persons insured by SHI (% Germany population)	70,011,718 (85.6)	69,803,236 (85.4)	69,637,277 (86.7)	69,704,323 (86.6)	69,861,165 (86.5)	70,289,808 (86.6)
No. persons in study sample (% Germany population)	3,646,060 (4.5)	3,708,501 (4.5)	3,985,981 (5.0)	3,982,716 (4.9)	3,977,676 (4.9)	3,793,331 (4.7)
Mean age, y	44.0	44.1	43.7	44.0	44.3	45.1
Sex ratio, F:M	1.03	1.03	1.04	1.04	1.04	1.04
No. case-patients with NTM-PD	85	96	106	106	104	126
M, no. (% case-patients with NTM-PD)	43 (50.6)	50 (52.1)	50 (47.2)	60 (56.6)	49 (47.1)	63 (50.0)
F, no. (% case-patients with NTM-PD)	42 (49.4)	46 (47.9)	56 (52.8)	46 (43.4)	55 (52.9)	63 (50.0)
Outpatient care, no. (% case-patients with NTM-PD)	74 (87.1)	79 (82.3)	80 (75.5)	88 (83.0)	85 (81.7)	104 (82.5)
Hospital care, no. (% case-patients with NTM-PD)	20 (23.5)	28 (29.2)	34 (32.1)	25 (23.6)	37 (35.6)	38 (30.2)
Outpatient and hospital care, no. (% Case-patients with NTM-PD)	9 (10.6)	11 (11.5)	8 (7.5)	7 (6.6)	18 (17.3)	16 (12.7)
Hospital but no outpatient care in the same year, no. (% case-patients who had hospital care)	11 (55.8)	17 (60.7)	26 (76.5)	18 (72.0)	19 (51.4)	22 (57.9)
Mean age of case-patients with NTM-PD, y (SD)	60.3 (20.0)	57.3 (22.2)	55.0 (23.8)	55.5 (25.4)	59.7 (22.6)	61.0 (20.4)
M	58.7 (20.1)†	55.3 (21.3)†	57.6 (20.0)†	55.2 (25.3)†	54.8 (24.6)‡	58.2 (19.3)†
F	62.1 (20.0)	59.4 (23.2)	52.7 (26.7)	56.0 (25.7)	64.1 (19.8)	63.7 (21.2)
Prevalence, rate/100,000 population, total (95% CI)	2.3 (1.87–2.87)	2.6 (2.11–3.15)	2.7 (2.19–3.20)	2.7 (2.19–3.21)	2.6 (2.15–3.16)	3.3 (2.78–3.94)
M	2.4 (1.76–3.20)	2.8 (2.06–3.59)	2.6 (1.92–3.34)	3.1 (2.36–3.92)	2.5 (1.88–3.29)	3.4 (2.63–4.31)
F	2.3 (1.65–3.03)	2.4 (1.81–3.22)	2.8 (2.10–3.56)	2.3 (1.68–3.0)	2.7 (2.07–3.51)	3.3 (2.53–4.14)
Projected total number of case-patients with NTM-PD in Germany	1,907	2,116	2,136	2,143	2,112	2,697

*SHI, statutory health insurance; NTM-PD, nontuberculous mycobacterial pulmonary disease. †Mean age was not significantly different among male and female patients: 2009, p = 0.44; 2010, p = 0.37; 2011, p = 0.28; 2012, p = 0.87; 2014, p = 0.13. ‡p = 0.038 for mean age between male and female patients.

**Table 2:** Most common and relevant comorbidities among case-patients with nontuberculous mycobacterial pulmonary disease, Germany, 2009–2014*

ICD-10 codes	Diagnosis	No. (%) patients					
		2009	2010	2011	2012	2013	2014
A31.0	NTM-PD	85	96	106	106	104	126
J43–44	COPD and emphysema	53 (62.4)	67 (69.8)	84 (79.2)	67 (63.2)	76 (73.1)	87 (69.0)
J40–42	Chronic or unspecified bronchitis	24 (28.2)	26 (27.1)	34 (32.1)	28 (26.4)	44 (42.3)	41 (32.5)
E10–11	Diabetes mellitus, type 1 and type 2	45 (52.9)	28 (29.2)	15 (14.2)	20 (18.9)	21 (20.2)	28 (22.2)
J09–18	Influenza and pneumonia	21 (24.7)	17 (17.7)	28 (26.4)	24 (22.6)	35 (33.7)	29 (23.0)
M80–81	Osteoporosis	18 (21.2)	20 (20.8)	27 (25.5)	23 (21.7)	23 (22.1)	26 (20.6)
J45	Asthma	18 (21.2)	25 (26.0)	31 (29.2)	22 (20.8)	23 (22.1)	34 (27.0)
J96	Respiratory failure	16 (18.8)	21 (21.9)	25 (23.6)	20 (18.9)	23 (22.1)	33 (26.2)
J20–22	Acute bronchitis and bronchiolitis	8 (9.4)	17 (17.7)	17 (16.0)	27 (25.5)	18 (17.3)	26 (20.6)
A15–19	Tuberculosis	12 (14.1)	14 (14.6)	16 (15.1)	21 (19.8)	25 (24.0)	17 (13.5)
F17	Tobacco use	13 (15.3)	17 (17.7)	17 (16.0)	16 (15.1)	14 (13.5)	16 (12.7)
K21	Gastro-esophageal reflux disease	13 (15.3)	15 (15.6)	18 (17.0)	14 (13.2)	17 (16.3)	20 (15.9)
J47	Bronchiectasis	6 (7.1)	9 (9.4)	9 (8.5)	7 (6.6)	16 (15.4)	23 (18.3)
C34	Lung cancer	3 (3.5)	4 (4.2)	7 (6.6)	9 (8.5)	5 (4.8)	13 (10.3)
M05–06	Rheumatoid arthritis	3 (3.5)	11 (11.5)	8 (7.5)	4 (3.8)	2 (1.9)	9 (7.1)
B20–24	Human immunodeficiency virus	1 (1.2)	2 (2.1)	5 (4.7)	4 (3.8)	5 (4.8)	5 (4.0)
D90	Immunosuppression	0	1 (1.0)	2 (1.9)	1 (0.9)	2 (1.9)	1 (0.8)
Z94	Transplant organ and tissue status	1 (1.2)	1 (1.0)	1 (0.9)	0	2 (1.9)	2 (1.6)
E84	Cystic fibrosis	0	1 (1.0)	0	0	2 (1.9)	2 (1.6)

*ICD-10, International Classification of Diseases, 10th revision; NTM-PD, nontuberculous mycobacterial pulmonary disease; COPD, chronic obstructive pulmonary disease.

We calculated rates, 95% CI, and differences between means using OpenEpi version 3.03a as previously described (*9*). Statistical significance was set to p<0.05. Accordingly, differences were considered insignificant if 95% CIs were overlapping. We obtained official census data from the German Federal Statistical Office (*7*). 

During 2009­–2014, we identified 85–126 case-patients with NTM-PD per year; distribution of sex was balanced (Table 1). Mean age was not significantly different between male and female patients except for in 2013, when the female patient ages were higher than those of the male patients (p = 0.038). Most (76%–87%) case-patients were treated in outpatient settings (Table 1). The most frequent concomitant diagnoses throughout the study period were chronic obstructive pulmonary disease and emphysema (COPD/emphysema [ICD-10 codes J43–J44]) in 62%–79% of the case-patients (Table 2).

**Figure 1:**
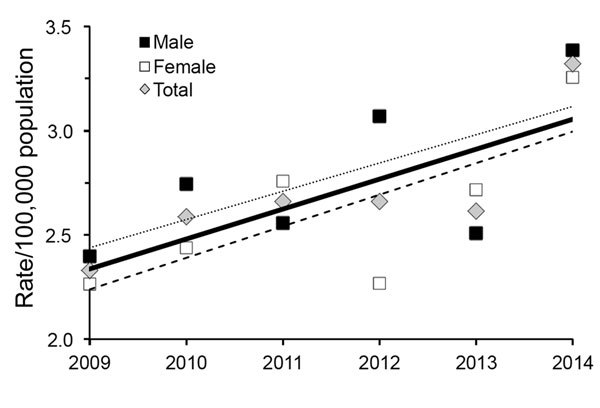
Trends in annual prevalence of nontuberculous mycobacterial pulmonary disease by sex and year, Germany, 2009–2014. Solid trend line indicates overall prevalence; dotted linear trend line, male prevelance; dashed linear trend line, female prevalence.

**Figure 2:**
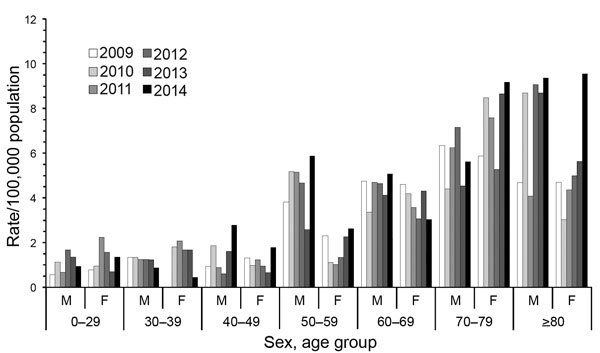
Prevalence rates of nontuberculous mycobacterial pulmonary disease, by age group, sex, and year, Germany, 2009–2014.

During 2009–2014, the annual overall prevalence rate increased from 2.3 (95% CI 1.87–2.87) to 3.3 (95% CI 2.78–3.94) per 100,000 population (Figure 1), and the corresponding projected total number of case-patients with NTM-PD in Germany increased from 1,907 to 2,697. Overall, annual rates did not differ substantively between male and female patients (Table 1). We observed the highest prevalence rates among case-patients >50 years of age, in particular among those ≥80 years of age in 2014 (9.4 [95% CI 4.35–17.78] cases/100,000 population for men and 9.6 [95% CI 5.44–15.65] cases/100,000 population for women; Figure 2).

## Conclusions

We found an increasing annual prevalence rate of NTM-PD in Germany during 2009–2014, from 2.3 to 3.3 cases/100,000 population. These rates are consistent with the average annual hospitalization rate of 0.9/100,000 population observed for 2005–2011 (*6*) but are considerably lower than the rate of 6.5/100,000 population that was recently estimated for Germany by an expert panel by using a 2-round Delphi consensus-building method (*10*). A study in the United States documented an overall prevalence of 5.5 cases/100,000 person-years and a prevalence rate of 27 and 57/100,000 population among case-patients >60 and >80 years of age, respectively, during 2004–2006 (*4*). This rate is ≈5 times higher than our findings of 6.5 and 9.5/100,000 population among the same age groups in Germany during 2014. In contrast to the US study, which found that NTM-PD was more frequent among women, we found no substantive differences concerning annual overall prevalence rates related to sex.

Several implications arise from our findings. Our analysis suggests that NTM-PD remains a rare disease in Germany, with a prevalence rate below the European cutoff of 5 cases/10, 000 population (*11*) and less than half the prevalence of tuberculosis (≈2,700 versus 6,300 prevalent cases in 2014) (*12*). Furthermore, in agreement with recent data from the United States (*5*), we found that chronic airway diseases (e.g., COPD/emphysema and bronchiectasis) were present in most case-patients diagnosed with NTM-PD (<79% and <18%, respectively). Hence, case-patients with these diseases, in particular those on long-term and high-dose inhaled corticosteroid therapy (*13*), should be considered for NTM-PD screening.

The data we collected indicate that most NTM-PD illness in Germany is managed in outpatient care (Table 1). In addition, our data show that a substantial proportion of case-patients had acute conditions (influenza and pneumonia, <34%) when concomitantly diagnosed with NTM-PD. We surmise that these case-patients are at risk for not being monitored for NTM-PD because >50% of them did not return as outpatients with the diagnosis of NTM-PD in the same year (Table 1). If this is the case, this finding emphasizes the need for continuing medical education on NTM-PD to increase clinical awareness.

Our study has limitations. First, the annual overall numbers of samples from case-patients in this study in which NTM-PD was diagnosed were low. Second, our findings are based on ICD-10 diagnosis codes and therefore are likely to underestimate the overall prevalence of NTM-PD, in particular when considering the low percentage of case-patients receiving American Thoracic Society/Infectious Diseases Society of America guideline-compliant treatment for NTM-PD (*14*). ICD-10 codes are primarily intended for reimbursement purposes; they lack validation for NTM-PD within the healthcare system of Germany and have unknown sensitivity and specificity for the use of the ICD-10 diagnostic code for NTM-PD. Moreover, assignment of ICD-10 codes does not require compliance with the American Thoracic Society/Infectious Diseases Society of America diagnostic criteria for NTM-PD (*1*), and the codes do not provide details on isolated mycobacterial species. Last, we were unable to account for regional differences across Germany. The eastern parts of Germany are slightly underrepresented in the Health Risk Institute database (*8*); therefore, our findings may not fully apply to all regions, and they may not fully apply to cohorts outside the German statutory health insurance system.

In conclusion, the annual prevalence rate of NTM-PD and, accordingly, the projected total number of cases in Germany increased during 2009–2014. Increased clinical awareness and additional reliable data on the epidemiology of NTM-PD are urgently needed, in particular with respect to COPD/emphysema. These goals could be met if NTM-PD were a notifiable disease or comprehensive disease-specific registries were established (*15*).
